# Inhibition of glucocorticoid-induced alteration of vimentin by a glucocorticoid receptor antagonist RU486 in the organ-cultured rat lens

**Published:** 2011-01-06

**Authors:** Guo-Li Xie, Hong Yan, Zi-Fan Lu

**Affiliations:** 1 Department of Ophthalmology, Tangdu Hospital, Fourth Military Medical University, Xi’an 710038, China; 2 Department of Biochemistry, Institute of Molecular Biology, Fourth Military Medical University, Xi’an 710032, China

## Abstract

**Purpose:**

Long-term application of glucocorticoids as a treatment for conditions such as allergy, autoimmune diseases, and transplantation presents a high risk of development of steroid-induced cataract. The presence of a functional glucocorticoid receptor (GR) in human and rat lens epithelial cells suggests a direct and specific targeting of these lens cells by glucocorticoids. One important cytoskeletal protein in lens epithelial cells is vimentin, which plays an important role in maintaining the normal lens morphology and function. Previous studies have shown that vimentin is involved in signal transduction, changes in cell structure and differentiation, and apoptosis. Based on a model of steroid-induced cataract from our previous study, the present study focuses on whether changes in vimentin can be induced in vitro through specific GR activation in glucocorticoid-induced cataracts of the rat lens.

**Methods:**

Clear rat lenses, cultured in vitro, were treated with or without dexamethasone (Dex) or RU486 (a glucocorticoid receptor antagonist). Lenses were cultured for 7 days at 37 °C under 5% CO_2_, and were observed daily with an inverted microscope. Changes in morphology were followed by Hematoxylin-eosin (HE) staining, transmission electron microscopy, and immunohistochemistry. The expression of vimentin mRNA and protein was examined by reverse transcription polymerase chain reaction (RT–PCR) and western blot analysis, respectively, in the capsule-epithelium and fiber tissue of the lenses.

**Results:**

Opacity was obviously present at day 7 in the Dex group. The lenses of the untreated group and the RU486+Dex group remained transparent throughout the incubation. Electron microscopy showed an orderly arrangement of fiber cells and normal cell junctions in the control group and the RU486+Dex group. However, in the Dex group, fiber cells were disarranged and the cell-cell junctions exhibited lacunae. The expression of vimentin protein in the lens capsule-epithelium and fiber tissue decreased in the Dex-treated group, but normal expression of vimentin mRNA was maintained.

**Conclusions:**

These results suggest that the GR-mediated reduction in vimentin may be involved in the formation of steroid-induced cataract.

## Introduction

Glucocorticoids are steroid hormones that are commonly employed as treatments for conditions such as allergy, autoimmune diseases, and transplantation. One established complication and side effect of prolonged glucocorticoid therapy is the formation of posterior subcapsular cataract characterized by nucleated epithelial cells in the posterior region of the lens [1-8]. The exact mechanism of glucocorticoid cataract formation is still unknown.

Glucocorticoids have been proposed to act on the lens through mechanisms involving binding of glucocorticoids to the hepatic glucocorticoid receptor (GR) [9-13], non-specific glucocorticoid binding in the lens [14-16], and binding to a membrane receptor [17]. Glucocorticoids have also been demonstrated in the aqueous humor [18,19]. Importantly, recent studies have provided evidence suggesting that rat and human lens epithelial cells contain the classic active GR, which verified a direct and specific mechanism of action [20-22], although this conclusion may not be valid in other species [16]. Glucocorticoids may also indirectly affect the lens through responses of other cells within the ocular compartment and/or through effects on cells at more remote locations. These remote effects could then modulate the intraocular levels of growth factors that normally control lens development and homeostasis [23].

Dexamethasone, one of the glucocorticoids, has a great affinity for the GR [24-26]. It is interesting to note that a high-dose prolonged dexamethasone (Dex) treatment of the rat lens resulted in a decrease in expression of cadherin protein, which is involved in cell adhesion [27]. Our recent study also showed that Dex treatment of rat lens resulted in a reduction in the activity and the protein and mRNA levels of the Na^+^,K^+^-ATPase, an important transport protein in the lens [28]. This finding raised the question whether glucocorticoids can induce changes in cytoskeletal proteins involved in cataract formation through the specific GR activation.

Vimentin is an important cytoskeletal protein in the epithelial cells of the lens, where it aids in maintaining normal lens morphology and function [29]. It is a highly conserved type III intermediate filament and is widely distributed in mesenchymal tissue, including the eye lens. Vimentin displays a typical tripartite domain structure with a central α-helical rod-domain flanked by non-helical amino- and C-terminal domains [30]. The vimentin network is present in both epithelial cells and some fiber cells of the lens. Fiber cells are vimentin positive up to a specific point 2–3 mm in from the lens capsule, after which the vimentin signal is drastically reduced [31]. However, vimentin appears to be absent from the older fiber cells, as indicated by both biochemical and immunocytochemical tests [32,33].

Recent evidence has shown that vimentin expression is related to glucocorticoid and its receptor. Vimentin determines cytoplasmic accumulation of the glucocorticoid receptor [34]. Oral steroid therapy has been shown to inhibit vimentin expression in densely packed spindle cells of Kaposiform haemangioendothelioma on the conjunctiva of the upper eyelid [35]. Dexamethasone prevents vimentin expression in the rabbit basilar artery after subarachnoid hemorrhage [36]. Overexpression of the chicken vimentin transgene in the mouse inhibits normal lens fiber cell differentiation and leads to cataract formation, suggesting that changes in vimentin expression are part of the mechanism underlying cataract formation [37,38].

Therefore, vimentin may interfere directly or indirectly with the proper cell differentiation processes in the lens, so that correct regulation of vimentin expression is essential for optical clarity. Changes in vimentin may affect the function of lens epithelial cells, which may further affect the transparency of the lens. This study evaluated whether glucocorticoid treatment can induce changes in vimentin through specific GR activation in glucocorticoid-induced cataracts of the rat lens.

## Methods

### Materials

Dexamethasone, RU486, Dulbecco’s MEM, and BSA were obtained from Sigma Chemical Company (Santa Clara, CA). Sprague-Dawley rats were provided by Animal Laboratories of the Fourth Military Medical University (Xi'an, China). Primary vimentin antibody was purchased from Epitomics Company (Burlingame, CA). Primary β-Actin antibody was obtained from Sigma Chemical Company. All other chemicals and solvents were of analytical grade and were obtained from local companies.

### Lens incubation and separation

All animal procedures conformed to the ARVO Statement for the use of Animals in Ophthalmic and Vision Research. For rat lens culture, eyes from three week old Sprague-Dawley female rats weighing 40–50 g were enucleated and placed in Dulbecco’s MEM (Sigma) containing BSA (Sigma), and antibiotic solution (100 U/ ml penicillin, 100 µg/ml streptomycin, and 0.25 µg/ml amphotericin B). The conditions for rat lens culture were as reported previously [27,39]. The eyes were opened posteriorly to avoid damaging the lens and pressure was applied to the anterior segment to expel the lens using plastic-coated forceps and fine scissors. Lenses were immediately transferred into culture medium, which was changed daily. Approximately 24 h after the preparation of organ cultures, clear lenses were selected, and dexamethasone (Sigma) was added to a final concentration of 5 µM [40]. RU486 (Sigma), a GR antagonist, and dexamethasone were added to the culture medium at a final concentration of 5 µM [27] to block glucocorticoid signaling. Lenses were cultured for 7 days at 37 °C under 5% CO_2,_ and were observed daily under a stereomicroscope and photographed. In a preliminary study, the effect of RU486 itself on the lens transparency was checked during 7 days incubation.

At the end of the culture period, the capsule-epithelium and the fiber tissue of lenses were removed by gently making an incision near the equator of the lens and peeling back the capsule-epithelium using fine forceps [41]. To slow proteolysis and mRNA breakdown, tissues were kept chilled during dissection. The separated lens tissues were frozen immediately in liquid nitrogen and stored at –70 °C.

### Observation of lens transparency

Whole rat lenses cultured in vitro were randomly divided into three groups: control group (DMEM), dexamethasone group (DMEM+5µM dexamethasone), and RU486+Dex group (DMEM+5µM dexamethasone+5µM RU486). Following 7 days of incubation, lenses were photographed daily with a stereomicroscope and graded for the development of opacity on a scale from 0 to 4 according to Mathur’s method, where 5 stages were given according to the lens clarity [27,40,42].

### Lens examination by electron microscopy

Two lenses from each group were examined by electron microscopy at day 7. Lenses were lightly fixed in 2.5% glutaraldehyde in 0.1M sodium phosphate buffer, pH 7.4, for 1.5 h at 4 °C. They were then fixed in glutaraldehyde/phosphate buffer overnight at 4 °C. After washing with phosphate buffer, the lenses were postfixed in 2% OsO_4_ in phosphate buffer for 1 h in an ice bath, rinsed with three changes of prechilled buffer and finally with distilled water. They were then stained with 2% aqueous uranyl acetate for 1 h in the dark. The lenses were dehydrated with increasing concentration (70%–100%) of ethanol and infiltrated with a mixture of propylene oxide/Epon-Aradite (Embed-812; EMS, Washington, PA) and embedded in Epon-Araldite. Ultrathin tissue sections (50–60 nm in thickness) were stained with 2% aqueous uranyl acetate for 30 min at 60 °C, rinsed with methanol and water, and stained with 0.3% lead citrate for 10 min at 60 °C. Sections were examined with a JEM-100SX (JEOL, Tokyo, Japan) electron microscope at 80 kV and at 10,000 fold magnification.

### Analysis of vimentin by western blotting

The lens capsule-epithelium and fiber tissues were homogenized separately in 1 ml of extraction buffer composed of 6 mM phosphate buffer (pH 7.2) containing 100 mM KCl, 5 mM MgCl_2_, 10 mM 2-mercaptoethanol plus 1 mM EGTA, 1 mM EDTA, 10 µM N-ethylmaleimide, 200 µM phenylmethylsulphonyl fluoride, and 5 µM E64 to prevent proteolysis during preparation. The homogenate was centrifuged at 12,000× g for 30 min to separate the soluble from insoluble proteins. The pellet was washed three times by resuspension in 1 ml extraction buffer and centrifugation. The washed insoluble pellet from the lens homogenate was separated into urea-soluble and urea-insoluble fractions by extraction in 8 M urea followed by centrifugation (12 000× g for 30 min). The final supernatant of the urea-soluble fraction was resuspended in extraction buffer, frozen in liquid nitrogen, and stored at −70 °C.

Proteins of the urea-soluble fraction of lens samples were separated by electrophoresis on a 12% sodium dodecyl sulfate-polyacrylamide gel using the Laemmli buffer system. Lens membrane protein samples (80 µg) were applied to specified lanes of the gel. The separated proteins were transferred electrophoretically to nitrocellullose at 100 V for 60 min in transfer buffer containing Tris (25 mM), glycine (192 mM), and 10% methanol at pH 8.3. Blots were incubated for 2 h at room temperature in TBS containing 0.1% Tween-20 (TBST) and 5% nonfat dried milk. They were then incubated overnight at 4 °C with the primary vimentin antibody (Epitomics), diluted in TBST, according to the manufacturer’s recommendations. Rabbit monoclonal vimentin and mouse monoclonal anti-β-Actin (Sigma) antibodies were used to detect the corresponding proteins. After washing three times with TBST, membranes were incubated for 1 h with horseradish peroxidase-coupled secondary antibodies (1:1,000 dilution; Santa Cruz Biotechnology, Santa Cruz, CA) at room temperature. Images were detected with the ECL kit (Pierce, Rockford, IL). The scanned images were quantified using Kodak Digital Science one-dimensional software (Eastman Kodak Co., Rochester, NY).

### HE stain and immunohistochemistry analysis for vimentin

Flat preparations of the equatorial capsule (containing adherent epithelial cells) were prepared by bisecting the lens. These were fixed for 24 h at room temperature in 4% paraformaldehyde in PBS. After fixation and paraffin-embedding, 5 µm thick tissue sections were obtained, and then were dehydrated with a graded series of increasing ethanol concentrations. The samples were embedded in Epon mixture. For each sample, the section was stained with hematoxylin-eosin. For immunohistochemistry analysis, the sample sections were blocked in PBS containing 5% goat serum and 0.1% Triton X-100, and incubated overnight with monoclonal anti-vimentin (Epitomics) at a dilution of 1:200. Samples were then incubated for 1 h with rhodamine-conjugated goat anti-rabbit IgG antibody (Zymed, San Diego, CA) at a dilution of 1:500. Histological images were photographed with a microscope (BX50; Olympus, Tokyo, Japan) and a digital camera (Nikon, Tokyo, Japan).

### Reverse transcription polymerase chain reaction (RT–PCR) analysis

Total RNA was prepared from the lens capsule-epithelium and fiber tissue with extraction reagent (Trizol; Invitrogen, Carlsbad, CA). Total RNA (2 µg) was used for reverse transcription with reverse transcriptase. PCR amplification was performed with reverse-transcribed DNA, 10 µM specific primers, 0.2 mM dNTP, and 1 mM magnesium chloride. The reaction was performed for 28 cycles of denaturation at 95 °C for 20 s, annealing at 54 °C for 20 s, and extension at 72 °C for 20 s. The PCR primer sequences and the sizes of the amplified products for each cDNA were as follows: vimentin, 5′-GCA AAG CAG GAG TCA AAC GA-3′ and 5′-CGT TTC GTC CTC AGT TTG CT-3′, 378 bp; and β-actin, 5′-AGG CCA ACC GCG AAG ATG ACC-3′ and 5′-GAA GTC CAG GGC GAC GTA GCA C-3′, 350 bp.

### Statistical analysis

The statistical significance of differences was assessed with Kruskal–Wallis H test using SPSS (Version 12.0) software. Results of opacification are presented as median values and range.

## Results

### Steroid-induced opacity in cultured lens and inhibition by RU486

To observe the effects of glucocorticoid on the lens, as previously reported [27,28,40], rat lenses were isolated and treated in organ culture with dexamethasone. In this study, mist-like opacity of the lenses was observed as early as 5 days in the dexamethasone (Dex) group (Table1). The opacity was more obvious at day 7. The lenses of the untreated group and the RU486+Dex group remained transparent during the incubation period (Table1).

**Table 1:** Opacification of rat lenses analyzed by ocular staging.

** **	**Incubation days**						
**Groups**	**1**	**2**	**3**	**4**	**5**	**6**	**7**
A	0 (0-0)	0 (0-0)	0 (0-0)	0 (0-0)	0 (0-0)	0 (0-0)	0 (0-0)
B	0 (0-0)	0 (0-0)	0 (0-0)	0 (0-0)	0 (0-1)^a^	1 (0-1)^a^	1 (0-1)^b^
C	0 (0-0)	0 (0-0)	0 (0-0)	0 (0-0)	0 (0-0)^c^	0 (0-0)^c^	0 (0-0)^d^

Lens opacification was assessed in control rat lenses or lenses treated with dexamethasone (Dex) or a combination of RU486 and dexamethasone (Ru486+Dex). Digital image analysis and ocular staging were performed by three independent observers. Following 7 days of incubation, lenses in the three groups were photographed daily with a stereomicroscope and graded for the development of opacity on a scale from 0 to 4, according to Mathur’s method, where 5 stages were given according to the lens clarity (Stage 0=clear lens, stage 1=lens with mist-like opacity, stage 2=lens with visible demarcation between nuclear and cortical regions, stage 3=lens with light nuclear opacification, stage 4=lens with dense nuclear opacity, stage 5=whole lens opacity). A=Control group; B=Dex group; C=RU486+Dex group. Values are expressed as medians (range of the ocular staging). Statistical analysis was performed using the Kruskal–Wallis H test. ^a^p<0.05 B versus ^b^p<0.01 B versus A. ^c^p<0.05 C versus B. ^d^p<0.01 C versus B.

In preliminary experiments, the rat lenses remained transparent throughout the incubation period in the presence of 5 µM RU486 without Dex (data not shown), indicating that 5 µM RU486 itself did not affect the transparency and the metabolism of the lens. A similar result was reported in the cultured rat lens by Lyn et al. [27]. Therefore, in the experiments, an RU486-only group was not included.

### The morphology changes of the cultured lens

**Figure 1:**
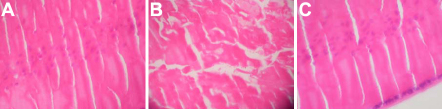
Morphological changes of the equatorial capsule (containing adherent epithelial cells) of the cultured rat lens. **A**: Control group; **B**: Dex group; **C**: RU486+Dex group. HE, original magnification 200×. A disordered arrangement of fiber cells was induced by treatment with dexamethasone (Dex) compared to the control. In the group treated with RU486 and Dex (RU486+Dex), no disruption was observed, which suggested that the morphological changes induced by Dex were blocked by RU486.

HE stain showed an orderly arrangement of fiber cells in the control group (Figure 1A) and the RU486+Dex group (Figure 1C). However, in the Dex group, the regular arrangement of fiber cells was disrupted and the lenses exhibited expanded extracellular lacunae (Figure 1B).

### Ultrastructure of the cultured lens

**Figure 2:**
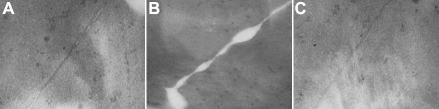
Morphological changes of the cultured rat lens. **A**: Control group; **B**: Dex group; **C**: RU486+Dex group. TEM, original magnification 120,000×). In the dexamethasone (Dex) group (**B**), the orderly arrangement of cell-junctions was disrupted compared with of the control group (**A**). In the group treated with both RU486 and Dex (RU486+Dex; **C**), no disruption was observed, which suggested that the morphological changes induced by Dex were blocked by RU486.

Transmission electron microscopy (TEM) showed an orderly arrangement and normal appearance of the cell junctions in the control group (Figure 2A) and the RU486+Dex group (Figure 2C). However, in the Dex group, the cell junction was disrupted and the lenses consisted of abnormal cells that exhibited vacuoles and expanded extracellular lacunae (Figure 2B).

### Effects of dexamethasone on vimentin protein levels in cultured rat lens

**Figure 3:**
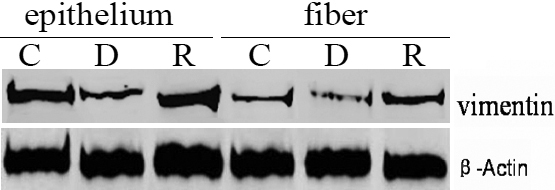
Levels of vimentin protein in rat lenses determined by western blot analysis. Vimentin protein levels in the whole lens were reduced in the group treated with dexamethasone (Dex; epithelium-D, fiber-D) compared with the control group (epithelium-C, fiber-C) and the group treated with Dex and RU486 (epithelium- R, fiber- R), which indicated RU486 prevented the reduced expression of vimentin protein induced by Dex . β-Actin was used as the control for protein loading. C: Control group; D: Dex group; R: RU486+Dex group.

We analyzed the expression of vimentin to determine whether it was involved in the observed morphological changes. Western blotting showed that vimentin protein levels in the capsule-epithelium and fiber tissue of the lenses were reduced in the Dex group (Figure 3, epithelium-D, fiber-D) compared with of the lens samples of the control group (Figure 3, epithelium-C, fiber-C) and of the RU486+Dex group (Figure 3, epithelium-R, fiber-R).

### Expression of vimentin in organ-cultured rat lens by immunohistochemistry analysis

**Figure 4:**
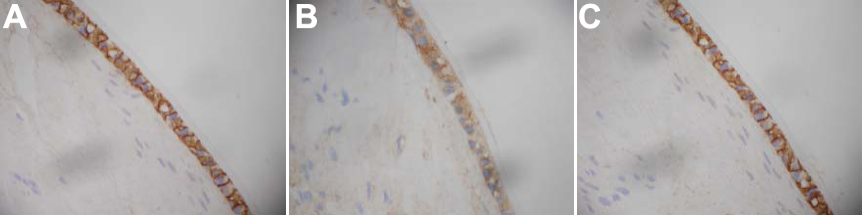
Vimentin protein expression in rat lenses. Vimentin protein level was reduced in the dexamethasone-treated (Dex) group (**B**) compared with of the control group (A) and the group treated with both RU486 and Dex (RU486+Dex; **C**). Magnification 400×.

Immunohistochemistry analysis showed that in the equatorial capsule (containing adherent epithelial cells), the levels of vimentin protein were reduced in the Dex group (Figure 4B) compared with the control group (Figure 4A) and the RU486+Dex group (Figure 4C).

### RT–PCR analysis

**Figure 5:**
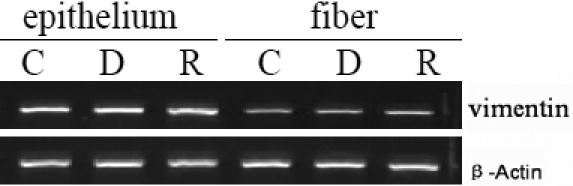
Reverse transcription polymerase chain reaction (RT–PCR) analysis of vimentinm RNA expression in the rat lens. The expression of vimentin mRNA was similar in all three groups, suggesting that GR translocation was indirectly involved in the changes in vimentin observed at the protein level. β-Actin was used as the control for protein loading. C: Control group; D: dexamethasone treated (Dex) group; R: group treated with both RU486 and Dex (RU486+Dex group).

The expression of vimentin mRNA, determined by RT–PCR, was similar in the capsule-epithelium and fiber tissue of the lenses of all three groups (Figure 5).

## Discussion

In this study, we have demonstrated that RU486 can prevent the reduction of vimentin protein expression and the changes in lens morphology induced by Dex. These results suggested that it is the GR that takes part in the changes in vimentin expression levels in the rat lens. This is the first report to demonstrate that the cataract induced by dexamethasone is associated with a reduced expression of vimentin protein but not expression of vimentin mRNA.

A mist-like opacity of the lenses was observed in the Dex group, whereas the lenses of the untreated group and the RU486+Dex group remained transparent throughout the incubation period, indicating that dexamethasone-induced lens opacification was inhibited by RU486.

Vimentin, as one of major cytoskeletal proteins in the lens, is expressed throughout the epithelium and elongating fiber cells of the adult mammalian lens [32,43,44]. Vimentin protein and mRNA are more abundant in the epithelium and in younger elongating fiber cells with a life-long lasting morphology [45]. By conventional immunocytochemical approaches, vimentin has been reported to be absent from, or at least greatly reduced in, the deeper cortical fiber cells [32,43,44]. Our current results showed that vimentin was highly expressed at both the protein and mRNA levels in the epithelium and to a lesser extent in the fiber mass of the lens, composed of the partial cortex and nucleus.

The precise role of vimentin in the biology of the lens is not known. However, previous studies showed that the changes of vimentin expression were involved in cataract formation. Capetanaki et al. [37] reported that overexpression of vimentin in transgenic mice inhibited normal fiber cell differentiation and led to cataract formation. Vimentin could interfere either directly or indirectly with the normal cell differentiation processes,so that correct regulation of vimentin expression may be essential for optical clarity. Previous studies have reported that changes in vimentin expression were responsible for apoptosis [46-48]. Vimentin is seemingly responsible for connecting the nucleus with the plasma membrane [49-51] and aids in transport processes and signal transduction. Further studies have shown that vimentin is involved in signal transduction, changes in cell structure, cell differentiation, and apoptosis in lens epithelial cells [52-54]. Therefore, changes in vimentin may affect the function of lens epithelial cells, leading to altered transparency of the lens [55]. The exact mechanism and pathogenesis of a steroid induced cataract are still unclear.

The GR was detected in both rat and human lens via in situ hybridization [20,21]. Receptor specificity was determined by a specific GC antagonist, RU486.Therefore RU486 provides a way to evaluate receptor-mediated events in the steroid induced cataract [56,57]. It can inhibit Dex-induced decrease in receptor activity [57,58]. In considering the mechanism for glucocorticoid effect on the lens, it should be noted that RU486 blocked the changes in gene expression and protein metabolism in lens epithelium treated with dexamethasone. Previous studies have shown that the GR present in the rat lens epithelial cells is able to bind with the classic glucocorticoid response element (GRE) and modulate the expression of a target gene [59,60]. In our current study, a statistically significant decrease in the protein expression levels of vimentin was observed under the treatment of 5 µM dexamethasone, but not in the negative control. Furthermore, these changes of vimentin were inhibited when the lenses were co-treated with RU486. These results indicated that the lens GR was responsible for glucocorticoid induced changes of vimentin.

Glucocorticoids, which play important regulatory roles in cellular physiology, are generally accessible to the cell membrane through passive diffusion and are able to bind to GR [61]. Agonist ligand binding activates the receptor by induction of a conformational change, resulting in dissociation of heat shock proteins and hyperphosphorylation of the receptor [62]. The ligand-receptor complex translocates to the nucleus, dimerizes, and binds to a cis-acting element, GRE, typically present in the 5′ flanking region of the target genes [63]. The conformational change gives the receptor-hormone complex the ability to bind DNA and modulate the expression of target genes [64]. DNA microarray hybridization data obtained from cultured human lens epithelial cells exposed to glucocorticoids has indicated that a broad range of transcripts are up- or down-regulated compared to unexposed control cells [60,65].

Glucocorticoid treatment of human lens epithelial cells results in activation of the GR and subsequent modulation of target gene expression via the mitogen activated protein kinases (MAPKs) and phosphatidylinositol 3 kinase/protein kinase B (PI3K)/AKT) pathways [60,65]. Analysis of microarray data from primary cultures of human lens epithelial cells has suggested that the glucocorticoid-GR activation may affect many cellular functions, such as proliferation, differentiation, apoptosis, survival, or migration, through modulation of the MAPK and PI3K/AKT pathways [57]. Since the MAPK and PI3K/AKT pathways have been reported to play important roles in similar cellular functions in many cell types, including lens epithelial cells [66,67], it is possible that the changes in modulation of these pathways could lead to abnormal lens epithelial cell proliferation, differentiation, motility, survival, or apoptosis, all of which have been implicated in the formation of a steroid-induced cataract. This idea is supported by the fact that regulators of the pathways were reported to be modulated as early as 4h and as late as 48h after Dex treatment of lens epithelial cells [60,65]. Glucocorticoids may be involved in lens cell function and cataractogenesis through the inhibition of these signaling pathways.

It is also interesting to note that involvement of both the PI3K/AKT and MAPK pathways has been reported for vimentin expression and activity [68]. Our study shows that the vimentin protein level was reduced in the capsule-epithelium of the lenses of the Dex group compared with the control group and the RU486 group, which suggested that glucocorticoid-GR mediation may lead to a reduction in vimentin expression. It is possible that decreased vimentin expression may lead to abnormal lens epithelial cell differentiation, signal transduction, survival, or apoptosis, and therefore to the formation of a steroid induced cataract. However, no differences were observed in the mRNA expression levels in the three groups, which indicated that the reduced expression of vimentin at the protein level was not occurring through GR-mediated GRE effects, but probably was a function of the modulation of the MAPK and PI3K pathways via glucocorticoid-GR mediation. Nevertheless, further evidence is needed to verify exactly where the MARK and PI3K/AKT pathways come into play in the cataract formation in lens epithelial cells.

In summary, we have demonstrated that the development of opacity induced by dexamethasone in the rat lens requires GR-mediated signaling. This opacity may proceed through the migration of abnormal epithelial cells, which results in the separation of lens fiber cells. Future study will focus on the precise mechanisms underlying the GR-mediated reduction in vimentin by dexamethasone.
